# Relationship of Cannabis Use With Immediate, Delayed, and Working Memory: The Health and Retirement Study

**DOI:** 10.1093/geroni/igab046.2634

**Published:** 2021-12-17

**Authors:** Madison Maynard, Daniel Paulson, Michael Dunn, Robert Dvorak

**Affiliations:** University of Central Florida, Orlando, Florida, United States

## Abstract

Past research has examined relationship between cannabis use and cognition among adolescents and young adults, but less is known about older adults despite rapidly increasing recreational and therapeutic cannabis use by this demographic. These relationships were explored cross-sectionally using data from the 2018 wave of the Health and Retirement Study (HRS). Dependent variables included immediate and delayed memory (10-item word list) and working memory (serial sevens; range 0-5). Cannabis use was categorized as non-user (n=886), past-user (n=334), current moderate (<52 uses/year; n=36), and current heavy (52+ uses/year; n=92). Mean age was 67.59 years (range: 50-98, SD=10.76). The sample was predominantly female (59%), and Caucasian (67%). Uncontrolled analyses found that cannabis use group was associated with immediate memory (F=6.14, p<.001), delayed memory (F=3.75, p=.01), and working memory (F=6.91, p<.001). Analyses controlled for gender, education, age, and race found that cannabis use group was no longer associated with delayed memory (F=1.74, p=.16) or working memory (F=1.66, p=.17); however, cannabis use was associated with immediate memory (F=3.75, p=.01) in controlled analyses. Current heavy users’ (M=4.94, SE=.16) immediate memory worse than that of both non-users (M=5.48, SE=.06) and past users (M=5.49, SE=.09; p<.05 for both). Gender, education, age, and race significantly associated with immediate, delayed, and working memory, respectively (p<.05 for all). In conclusion, relative deficits in immediate memory, but not delayed memory or working memory, were associated with current heavy cannabis use among older adults. In combination with other findings, these results may inform development of safe-use guidelines for older adults.

